# The “Emotional Side” of Entrepreneurship: A Meta-Analysis of the Relation between Positive and Negative Affect and Entrepreneurial Performance

**DOI:** 10.3389/fpsyg.2017.00310

**Published:** 2017-03-13

**Authors:** Oana C. Fodor, Sebastian Pintea

**Affiliations:** Department of Psychology, Babes-Bolyai UniversityCluj-Napoca, Romania

**Keywords:** meta-analysis, positive affect, negative affect, entrepreneurial performance, entrepreneurship

## Abstract

The experience of work in an entrepreneurial context is saturated with emotional experiences. While the literature on the relation between affect and entrepreneurial performance (EP) is growing, there was no quantitative integration of the results so far. This study addresses this gap and meta-analytically integrates the results from 17 studies (*N* = 3810) in order to estimate the effect size for the relation between positive (PA) and negative affect (NA), on the one hand, and EP, on the other hand. The meta-analysis includes studies in English language, published until August 2016. The results indicate a significant positive relation between PA and EP, *r* = 0.18. The overall NA – EP relation was not significant, *r* = -0.12. Only state NA has a significant negative relation with EP (*r* = -0.16). The moderating role of several conceptual (i.e., emotion duration, integrality etc.), sample (i.e., gender, age, education) and methodological characteristics of the studies (i.e., type of measurements etc.) are explored and implications for future research are discussed.

## Introduction

The experience of work in an entrepreneurial context is saturated with affective experiences. Meeting an important deadline, pitching an idea to a business angel, deciding whether or not to follow through with an investment, negotiating with clients and suppliers are but a few examples of entrepreneurial work situations permeated by emotions. They also play a pivotal role in shaping human cognition, motivation and behavior (Judge and Larsen, 2001; Brief and Weiss, 2002; Barsade et al., 2003) and, consequently, they influence entrepreneurial performance (EP) (Baron, 2008; Gorgievski et al., 2010; Baron and Tang, 2011 etc.).

Research has thus proliferated in this area in the past few years (Delgado Garcia et al., 2015) and focused on exploring the relation between both positive and negative affect, on the one hand, and different measures of EP, on the other hand. Traditionally, a significant body of evidence has accumulated in support of a positive relation between positive affect (PA) and EP, as PA is associated with important cognitive (i.e., it increases cognitive flexibility and creativity) (Baron and Tang, 2011) and motivational benefits (i.e., it stimulates effort investment in dealing with entrepreneurial tasks (Foo et al., 2009). Alternatively, negative affect (NA) was generally associated with negative implications for EP as it has negative implications for cultivating rewarding social ties (Lucas and Diener, 2003), creativity (Hills et al., 1999) or adjusting to dynamic environments (Baron, 2008). However, the findings are far from consistent, as there is also evidence for the negative association between PA and EP. High levels of PA impede firm innovation and sales growth rate (e.g., Baron et al., 2011), for instance. Similarly, there is also evidence supporting the positive link between NA and behaviors conducive to performance (i.e., stimulating entrepreneurs to approach tasks that are immediately required) (e.g., Foo et al., 2009).

To our knowledge, so far there were no efforts to quantitatively integrate this line of research. Moreover, little is known about the differential effect of positive vs. negative emotional experiences or about the contextual and methodological contingencies that might condition the relation between PA and EP and NA and EP (Delgado Garcia et al., 2015).

In this study, we address this particular gap and meta-analytically integrate the results of 17 studies exploring the relation between positive and negative affective experiences (i.e., emotions, mood, dispositional affect) and EP. The contributions of the study are threefold. First, we determine the magnitude of the relation between affective experiences and the EP. Second, we disentangle between the effect of positive vs. negative affective experiences on EP, while also determining the direction of the relation (i.e., positive vs. negative). Third, we test the role of moderators pertaining to affect, level of analysis of the outcome, individual differences, and study quality in order to identify how the influence of affective experiences varies under different conditions. Based on our meta-analytic findings, we conclude by reflecting on the theoretical and methodological implications for the literature on affect and EP.

## Theoretical Development

### A Brief Look at Affective Experiences

In entrepreneurship literature, affect encompasses a broad range of phenomena ranging from affective dispositions, moods, and emotions, to different affect related abilities such as emotion regulation (i.e., the entrepreneur is able to manage his/her fear of losing and decides to invest), emotional labor and emotion intelligence. In this study we focus on entrepreneurial affect conceptualized as different types of subjective feelings experienced by the entrepreneur (i.e., dispositional affect, moods, emotions and passion) and the way they influence EP. Thus we distinguish affect from meta-emotional abilities (i.e., abilities to recognize and regulate one’s own or others’ affective states), which are excluded from our endeavor.

*Dispositional affect* refers to an affective personality or one’s tendency to experience positive vs. negative affect across situations and time (Watson et al., 1988; Barsade and Gibson, 2007). Positive affectivity (as a trait) is characterized by stable patterns of experiencing enthusiasm, pleasurable engagement and high energy, whereas negative affectivity (as a trait) is described by a tendency to experience distress, unpleasurable engagement and nervousness (Watson et al., 1988). *Moods* are diffuse affective states that arise in response to general stimuli (i.e., pleasant vs. unpleasant mood, feeling good or bad). They have little cognitive content (Forgas, 1995), are low in intensity and relatively enduring (Frijda, 1986; Barsade and Gibson, 2007). In contrast, *emotions* are intense emotional episodes, generated by a particular stimulus and shorter in duration (Frijda, 1986; Barsade and Gibson, 2007). Since emotions are strongly connected to an event, they are rich in cognitive content (i.e., fear arises in relation with a particular event, where consequences are potentially negative, yet uncertain).

*Passion* is a particular type of affective state connected with the entrepreneurial process. In line with Cardon et al. (2009), we conceptualize entrepreneurial passion as an intense positive emotion, directed toward typical activities that are linked to the entrepreneurial role identity. Passion comes with an important motivational effect such that it fosters task engagement and enables the entrepreneur to surpass the drawbacks in his/her activity.

### Entrepreneurial Performance

We follow Shane and Venkataraman (2000) and define entrepreneurship as the process of identifying and exploiting opportunities to produce goods and deliver services, with the goal of making profit. Thus, for the purpose of this research, EP is conceptualized as the extent to which entrepreneurs and their organizations fulfill goals such as: profitability, business growth and innovation (Hitt et al., 2001; Wang et al., 2004; Gorgievski et al., 2011). Thus, this study includes indicators such as profit margin for *profit*, growth in sales, revenue, number of employees (i.e., as indicator of number of jobs created) or market share for *business growth*, number, novelty and usefulness of innovations for *innovation*. Since the link between the company and the entrepreneurs’ actions is tight, we include in the conceptualization of EP the entrepreneurs’ volitional actions and behaviors that contribute directly to the achievement of these goals as well (in line with Campbell, 1990) and indicators such as goal realization and innovative behavior. In addition, we also introduce *subjective ratings of performance* such as satisfaction with the financial or overall business performance, as they are rooted in the actual economic performance of the firm (Dej, 2011; Gorgievski et al., 2014). However, we subsequently aim to disentangle the differential effects of the type (objective vs. subjective) and level of measurement of EP on the affect – EP relation by performing a moderators’ analysis detailed in the sections below.

### Affect and Entrepreneurial Performance – a Theoretical and Empirical Account

Affective experiences are increasingly being acknowledged as important drivers of performance in organizational settings (Barsade and Gibson, 2007; Shockley et al., 2012), in general, and of entrepreneurial success, in particular (Baron and Tang, 2011; Ho and Pollack, 2014), via their impact on cognitive and motivational processes that support organizational behavior.

It has been argued that entrepreneurship is even more emotionally laden as compared to other organizational processes and settings (Baron, 2008). A range or arguments support this claim. First, identifying and exploiting a business opportunity is associated with strong identity and emotional connections (i.e., passion) between entrepreneur and ‘the idea’ (Cardon et al., 2005). Metaphorically, entrepreneurship is thus described as ‘parent and child’ (Cardon et al., 2005, p. 24). Second, entrepreneurship involves high stakes, as well as strong commitment. Entrepreneurs invest substantial financial resources, time and effort in developing and exploiting their idea. Even small wins or loses are associated with a more intense emotional intrusion (Schindehutte et al., 2006). Third, entrepreneurial tasks are complex, marked by uncertainty and fast changing conditions that do not favor a reliance on pre-established routines. The *affect infusion model* (AIM) (Forgas, 1995; Forgas and George, 2001) postulates that it is particularly in these circumstances that entrepreneurs may overuse their ‘feelings’ as cues for further action (Baron, 2008). Therefore, our first research question is concerned with exploring:

RQ 1: How strong is the relation between affective experiences and the EP?

The affect domain has been dominated by a persistent debate regarding the dimensionality of affect (Watson and Tellegen, 1985; Russell and Carroll, 1999). However, in this meta-analysis we follow the conceptualization employed by Watson et al. (1988) who argue that affective experiences can be classified along two separate unipolar dimensions: positive affect (PA; or positive activation) and negative affect (NA; or negative activation), instead of a one bi-polar continuum. The main arguments supporting this claim concern: (1) the low correlations between PA and NA (Watson and Clark, 1997), (2) the findings indicating different correlates and correlates of different magnitudes for PA and NA (Kaplan et al., 2009) (for instance, PA is associated with extraversion, but not neuroticism, while NA is associated with neuroticism, but not extraversion; Russell and Carroll, 1999), and (3) the findings indicating that PA and NA operate via different underlying biological mechanisms (Watson et al., 1999). The following sections review evidence concerning the association between PA and EP, and NA and EP, respectively.

#### Positive Affect and Entrepreneurial Performance

Traditionally, organizational behavior literature has revolved around the *happy worker – productive worker* framework and, indeed, PA (emotions, moods, dispositional affect, passion) has been systematically linked with superior performance outcomes across various fields. Kaplan et al. (2009) meta-analytically integrated results concerning the relation between dispositional affect and performance and showed that trait PA is related with both task and extra-role performance (i.e., organizational citizenship behaviors such as helping co-workers, which further support organizational functioning and are conducive for performance; Brief and Motowidlo, 1986; Borman et al., 2001). Individuals who report experiencing positive emotional states more often are also rated as more effective at their jobs (Staw et al., 1994).

Research in entrepreneurial settings has recently produced parallel results, supporting the benefits associated with experiencing PA for both individual and company performance. For instance, positive dispositional affectivity is significantly related to the statement of a broad and ambitious set of goals and satisfaction with business performance (Delgado-Garcia et al., 2012). It enhances entrepreneurs’ effort on both present and future oriented entrepreneurial tasks (Foo et al., 2009), and stimulates cognitive flexibility and creativity (Baron and Tang, 2011) – important precursors of innovation as a measure of company performance. In addition, positive affective traits are associated with product innovation and increased sales rate up to an inflection point (Baron et al., 2011).

In a similar vein, Cardon and Kirk (2013) showed that entrepreneurial passion (i.e., intense positive feelings associated with identification with entrepreneurial activity) is an important predictor of sustained entrepreneurial action and mediates the effect of self-efficacy on the latter. Baum and Locke (2004) reported that passion is positively related with individual goal attainment and self-efficacy, which are further related with business growth. Drawing on the dualistic model of passion, Ho and Pollack (2014) provide support for a positive link between entrepreneurial harmonious passion (i.e., a controllable desire to engage in activity) and initiating contacts in the network (i.e., out-degree centrality), which, in turn, was associated with an increased financial performance for the firm. While Patel et al. (2015) reported a positive association between harmonious passion and job creation, especially under environmental complexity. To sum up, there is a consistent body of evidence in support of a positive relation between PA and EP at both individual and company level, via cognitive and motivational mechanisms.

Theoretically, the positive effect of PA can be explained by Fredrickson’s (2001) broaden-and-build theory that states that PA signals a benign environment that further encourages the entrepreneur to broaden her/his attention scope and invest more in exploring, creating and seizing opportunities. Overall, experiencing PA is associated with making better use of entrepreneurial resources (i.e., establishing more social contacts that allow an easy access to information or funding, for instance; Ireland et al., 2003; Lucas and Diener, 2003) and being more able to handle entrepreneurial tasks (Gartner et al., 1999).

An alternative explanation for the PA – EP link is derived from the approach-avoidance theories (Gray and McNaughton, 2000; Corr, 2013). In short, PA is considered to activate the Behavioral Approach System (BAS), an underlying neuropsychological system that triggers appetitive, reward seeking behaviors, which are aligned to the specifics of entrepreneurial tasks and conducive for performance.

In spite of the accumulating evidence in support of the beneficial effects of positive entrepreneurial affect, the link with performance outcomes is much more complex and several studies also reported detrimental effects. For instance, Baron and collaborators showed that too much dispositional PA is conducive to a decline in innovation and firm sales (Baron et al., 2011) or can lead to an optimism bias (Baron, 2007, 2008; Baron et al., 2012) which are detrimental for performance. The negative effect of PA on EP measures such as sales was explained by a reduction of the effectiveness of the entrepreneur’s persuasion efforts (i.e., too much PA might appear as insincere to the target/buyer, Sharma and Levy, 2003). The other possible mechanism is a motivational one: when experiencing positive affect, entrepreneurs might be motivated to disregard or downplay crucial but negative information coming from customers that might alter their affective state (Forgas, 2001). Failure to consider negative information, in turn, has negative implications for the capability of the firm to adapt when facing changing market conditions (Teece et al., 1997). In a similar vein, obsessive passion (i.e., an uncontrollable desire to engage in activity) has been associated with negative consequences for entrepreneurial individual performance and, consequently, for the company performance. For instance, Vallerand pointed out that obsessive passion is linked with a rigid persistence in tasks and this is associated with a reduced quality of interpersonal relationships (Vallerand et al., 2003), which are important for entrepreneurial success.

To sum up, contradictory evidence regarding the PA – EP relation is present and meta-analysis could shed light on the nature of this relation. However, in line with the increasing theoretical and empirical evidence reviewed above we hypothesize that:

Hypothesis 1: Positive affect is positively related with EP.

#### Negative Affect and Entrepreneurial Performance

In contrast to PA, empirical results show that both trait NA (Kaplan et al., 2009) and state NA (Kaplan et al., 2009; Shockley et al., 2012) have negative implications for performance, as they are positively linked to counterproductive work behaviors (i.e., CWBs). CWBs are negative behaviors (i.e., information hiding, theft, withdrawal behaviors, etc.) intentionally enacted by employees/ entrepreneurs, which impair the organization’s capacity to fulfill its goals and threaten its well-being (Spector and Fox, 2005).

Entrepreneurship research also provides a number of arguments in support of the negative association between NA and EP. In line with Fredrickson’s (2001) broaden-and-build theory, NA signals a malign environment and, as such, it is expected to trigger a narrower thought-action repertoire. That is, when experiencing NA, entrepreneurs would be less creative – which is associated with less opportunity recognition (Hills et al., 1999) –, would engage less in cultivating their network – which is crucial for resource acquisition (Lucas and Diener, 2003) and have difficulties to adjust to the dynamic environment they operate in (Baron, 2008). These claims are doubled by the postulates of the approach-avoidance theories (Gray and McNaughton, 2000; Corr, 2013) according to which NA activates the Behavioral Inhibition System (BIS), an underlying neuropsychological system that triggers avoidance rather than exploratory behaviors.

Empirically, entrepreneurship studies have shown that NA is associated with a narrow set of goals, a reduced satisfaction with business performance (Delgado-Garcia et al., 2012), and reduced business growth (Gorgievski et al., 2014). Poor mental health (i.e., depressed mood, strain etc.) of business owners also predicted a reduced economic performance of the firm over time (Gorgievski et al., 2000, 2010).

However, NA can also have positive effects for EP, as it stimulates entrepreneurs to approach tasks that are immediately required (Foo et al., 2009). In addition, negative moods seem to drive a more systematic information processing that proved to be beneficial for the idea selection phase, as part of the entrepreneurial creative process (Perry-Smith and Coff, 2011). In a similar vein, negative affect drives the selection of safer decision heuristics in the case of entrepreneurs (Fodor et al., 2016), as when experiencing affect appraised as negative, the negative valence spills over the decision situation and acts as a signal that the decision situation might include a threat to the achievement of desired goals (Schwarz and Clore, 1983). Such circumstances favor a more careful and error avoiding behavior (Fiedler, 2001) which is likely to foster performance.

Data on the NA – EP link is mixed and would benefit from meta-analytical integration. In line with the body of evidence reviewed above, we hypothesize that:

Hypothesis 2: Negative affect is negatively related with EP.

### Moderators of the Affect – Entrepreneurial Performance Relationship

In addition to the magnitude of the effect that affective states have upon EP, little is known about the contextual and methodological contingencies that might condition their influence (Delgado Garcia et al., 2015). As for the moderators, we included variables related to: (a) level of measurement for the outcome variable (individual vs. company level), as previous research postulated different magnitudes of the affect –performance relation based on the proximal or distal nature of the outcome (Baron et al., 2011); (b) affect characteristics (i.e., duration and embeddedness in the entrepreneurial process) as previous meta-analysis proved they modulate the influence of affect on various outcomes (i.e., decision making etc.) (Angie et al., 2011); (c) individual differences as they affect the generalizability of the findings and previous research proved they influence emotional reactivity (Gross and John, 2003; Martin and Ochsner, 2016); and (d) study quality, as features and quality of the research design have important implications when exploring the influence of emotional experiences on various outcomes (Parrott and Hertel, 1999; Angie et al., 2011).

#### Level of Measurement for the Outcome Variable: Individual vs. Company Level Performance

This moderating variable refers to the unit that the data regarding performance in the original study described: individual level performance (i.e., number of innovations generated and implemented by an entrepreneur) vs. company level performance (i.e., number of innovations implemented within the firm). While the link between the entrepreneur and his venture is tight as argued before, both affect and individual level performance pertain to the entrepreneur and the influence of one over the other is likely to be stronger, as compared to the influence of affect – as an individual level variable – on firm performance – as a venture level and more distal variable. In this latter case, it is probable that there are multiple mediating and moderating mechanisms that make the affect – EP (i.e., measured at the company level) relation more complex (Baum and Locke, 2004; Baron and Tang, 2011). Therefore, we argue that:

Hypothesis 3a: The positive association between positive affect and EP is stronger for individual performance as compared to company performance.
Hypothesis 3b: The negative association between negative affect and EP is stronger for individual performance as compared to company performance.

#### Affect Characteristics

By taking into consideration the durability, affect can be categorized as state or trait. State affect (i.e., moods and emotions) includes short-term and transient affective experiences that arise in response to an environmental trigger (i.e., internal or external). Trait affect (i.e., dispositional) refers to enduring affective patterns – a tendency to experience a certain type of affect (i.e., positive or negative) across situations (Watson and Clark, 1984; Barsade and Gibson, 2007). Theories that integrate the two (i.e., Affect Events Theory; Weiss and Cropanzano, 1996), postulate a mediator role for state affect in the relation between trait affect and various outcomes and, thus, indicate a more intimate link between state affect and outcomes. However, given that EP – as conceptualized in this paper – is the sum of numerous performance episodes across a longer time frame that ultimately lead to profit, growth, goal attainment etc., and the persistent vs. more temporary character of trait vs. state affect, we anticipate that trait affect will be more influential for EP.

Hypothesis 4a: The positive association between positive affect and EP is stronger for trait as compared to state affect.
Hypothesis 4b: The negative association between negative affect and EP is stronger for trait as compared to state affect.

Lastly, the extent to which affective experiences are related to the entrepreneurial task can also modulate the influence of affect on EP. For instance, affect can be embedded in the entrepreneurial process, in the sense that it is experienced in direct relation to the task itself (e.g., an entrepreneur who experiences passion for finding new opportunities). This is called integral affect (Angie et al., 2011). On the other hand, incidental emotional experiences are unrelated to the entrepreneurial task at hand. They encompass dispositional affect (e.g., trait anxiety), pre-existing mood states and emotions generated by events unrelated to the entrepreneurial tasks (e.g., sadness generated by losing a loved one). In some cases, incidental emotions have carry-over effects, in the sense that they are strong enough to influence cognitive processes unrelated to the original triggering event (Lerner and Keltner, 2000; Lerner and Tiedens, 2006). However, given the intimate connection between integral affective experiences and the entrepreneurial tasks, we anticipate that they will be more influential for EP, as compared to incidental affect.

Hypothesis 5a: The positive association between positive affect and EP is stronger for integral as compared to incidental affect.
Hypothesis 5b: The negative association between negative affect and EP is stronger for integral as compared to incidental affect.

#### Individual Differences As Moderators

The nature of the sample included in the study is important not only for the generalisability of the findings, but also for the way associated variables can influence the nature and magnitude of the relation between affect and EP. Gender is claimed to have an important role, as men and women show different patterns of emotional reactivity and emotion regulation (Bradley et al., 2001; Gross and John, 2003). Women, for instance, show greater physiological responses to emotional stimuli (Bradley et al., 2001; Wild et al., 2001), tend to express emotions to a greater extent than men (Grossman and Wood, 1993; Hess et al., 2000), and have better episodic emotional memory (Seidlitz and Diener, 1998). Thus, by generally being more emotional than men, women entrepreneurs might be more vulnerable to the intrusion of affect in the entrepreneurial process.

Emotion regulation refers to a person’s ability to manage or change an emotional state via various active strategies (i.e., suppression, reappraisal etc.). Developmental research on emotion regulation has proven that the ability to down-regulate affective experiences improves with age, as it depends on the maturation of different pre-frontal cortical regions, (Martin and Ochsner, 2016) and can be educated.

Therefore, by taking into consideration the evidence regarding the influence of individual differences on the affect – EP relation, we state that:

Hypothesis 6a, 7a, and 8a: The positive association between positive affect and EP is stronger for women (6a), for less educated (7a) and for younger (8a) entrepreneurs as compared to men, more educated and older entrepreneurs.
Hypothesis 6b, 7a, and 8a: The negative association between negative affect and EP is stronger for women (6a), for less educated (7a) and for younger (8a) entrepreneurs as compared to men, more educated and older entrepreneurs.

#### Study Quality As Moderator

The features and quality of the research design have important implications when exploring the influence of emotional experiences on various outcomes (Parrott and Hertel, 1999; Angie et al., 2011). We used two moderators for study quality. As the measurement format of the variables may introduce a certain bias (Parrott and Hertel, 1999), one of the moderators concerned whether the measurement of the entrepreneurial construct was objective or subjective. The second moderator we coded for is the ecological validity of the study design or the extent to which the studies were conducted in the laboratory and used artificial task such as simulations or vignettes (i.e., non-ecological design), or they were conducted in real life settings and captured the entrepreneurs’ experience on real, natural tasks (i.e., ecological design).

Based on these issues, our second research question refers to:

RQ2: How do variables related to study quality moderate the relation between affective experiences (positive and negative) on the entrepreneurial process?

## Materials and Methods

### Literature Search

To ensure a comprehensive search of relevant articles for performing the meta-analysis, we employed strategies such as: (a) searching the archives of journals known to publish research on the topic such as: *Entrepreneurship: Theory and Practice*, *Journal of Business Venturing, Journal of Small Business Management*; (b) searching Ebsco, ProQuest’s ABI/INFORM, Wiley Online Library, ScienceDirect, Emerald Fulltext, Sage, Web of Science – Social Science Citation Index databases; and (c) examining the reference list of previously identified relevant articles, especially recent theoretical reviews on the role of affect in entrepreneurship (Delgado Garcia et al., 2015). While conducting the database search, the following keywords and their combinations were used: *entrepreneur* (with derivates such as ‘entrepreneurial,’ ‘entrepreneurship’) and *performance* (with derivates such as ‘venture/firm/company/business performance’) and *affect* (with derivates such as ‘affective,’ ‘affectivity’), *mood* or *emotion* (with derivates such as ‘emotional’). The literature search started in 2015 and ended in August 2016, with no time limit in terms of the publication year of the manuscript and using English language.

In order to mitigate the potential bias of unpublished research, which tends to have smaller mean effect sizes or non-significant results (Lipsey and Wilson, 2001), we also conducted a manual search of abstracts and proceedings from relevant conferences.

### Inclusion and Exclusion Criteria

Three criteria were set for the inclusion of the studies in the meta-analysis. First, the studies were required to explore and include data regarding the relation between an experienced affective construct (i.e., emotion, mood, affect, passion) and EP (i.e., individual or venture performance). Thus, studies that examined affect as a consequence of the EP (i.e., the way business failure is likely to produce grief) or other antecedents of entrepreneurial affect were not included in the meta-analysis. Studies exploring the impact of affect related constructs (i.e., emotional intelligence, emotion regulation, emotional stability) on EP but not complying with the conceptualization provided in the study were also excluded from further analysis. Second, the studies were required to employ an experimental, cross-sectional or a longitudinal design. Qualitative studies, conceptual papers, theoretical reviews and editorial notes were excluded. Third, the articles were required to provide sufficient statistical information to compute effect sizes.

### Data Set and Coding Procedure

After a preliminary examination of the titles and abstracts, around 50 articles complied with the first criterion of inclusion in the meta-analysis; that is, they seemed to explore the relation between an affective variable and EP and were considered for further analysis. After reading the full-text and applying all the inclusion-exclusion criteria (1 – the study explored the relation between an affective construct and EP outcomes as conceptualized in the meta-analysis, 2 – the study was not qualitative, theoretical review or an editorial note, and 3 – the study included sufficient statistical information to compute effect sizes), a final sample of 17 studies (*N* = 3810 participants) was retained for the quantitative analysis, yielding 76 effect sizes. **Table 1** provides a summary of the studies included in the meta-analysis.

**Table 1:** Descriptive summary of the studies included in the meta-analysis.

Study	Entrepreneurial construct	Emotion construct	Sample	*N*	No. of effect sizes
Baron and Tang, 2011	Dynamism, entrepreneurial creativity, firm sales, no. of innovations, radicalism of innovation	Positive affect	E	99	4
Baron and Tang, 2011	Firm age, firm net worth, firm size, no of innovations, sales growth rate	Positive affect	E	157	3
Baum and Locke, 2004	Company size, venture growth	Passion	E	229	1
Cardon and Kirk, 2013	Entrepreneurial persistence, firm sales	Passion for inventing, founding and developing	E	129	3
Delgado-Garcia et al., 2012	Business age, business size, financial and non-financial goals, satisfaction with business performance, short term goals	Personal negative affect, environmental negative affect, positive affect	E	335	2
Deniz et al., 2011	Financial performance, innovative performance	Fear of work, fear of unknown, fear of non-monetary support, fear of uncertainty, fear of legal issues	E	225	10
Gorgievski et al., 2014	Business growth, innovative behavior, subjective business performance	Positive affect, negative affect	E	180	6
Gorgievski et al., 2010	Objective financial situation, perceived financial problems	Distress	E	260	12
Gorgievski et al., 2000	Perceived financial problems	Perceived mental health (distress)	E	182	6
Ho and Pollack, 2014	Financial performance, organization size	Harmonious passion, obsessive passion, excitement	E	206	4
Ismail et al., 2016	Entrepreneurial success	Entrepreneurial passion	E	246	1
Laguna et al., 2016	Goal realization	Goal related positive affect, goal related negative affect	E	246	4
Murnieks et al., 2014	Revenue growth, time spent on business	Passion	E	204	1
Patel et al., 2015	Jobs created	Harmonious passion, obsessive passion	E	105	2
Perry-Smith and Coff, 2011	Creativity (novelty, number of unique ideas, usefulness)	Unactivated unpleasant mood, activated unpleasant mood, unactivated pleasant mood, activated pleasant mood	Non-E	187	12
Thorgren and Wincent, 2015	Venture turnover, venture result (profit or loss)	Harmonious passion, obsessive passion	E	704	4
Wincent and ÖRtqvist, 2011	Sales	Positive affect	E	116	1


*N, sample size in the study, No. of effect sizes, number of effect sizes retrieved from the study, E, entrepreneurs (the sample in the study included entrepreneurs), Non-E, non-entrepreneurs (the sample in the study included other participants, not entrepreneurs).*

Each study was coded for moderators referring to: (a) *level of measurement for the outcome* (i.e., individual vs. business performance); (b) *affect characteristics* such as: duration (i.e., state vs. trait affect), and whether the affective construct was related to the entrepreneurial process or not (i.e., integral vs. incidental); (c) *features of the study design*: whether the measurement of EP was objective or subjective and the ecological validity of the study design (i.e., ecological vs. non-ecological); and (d) *sample characteristics* such as: participants’ status (i.e., entrepreneurs vs. non-entrepreneurs), proportion of women, average age in the sample, and proportion of higher education. Other moderators pertaining to the sample were also initially considered and coded (i.e., industry, entrepreneurial experience etc.), but were later dropped from the analysis due to lack of information from the original studies.

To ensure coding consistency and construct validity, the coding scheme was jointly developed by the authors in line with the conceptual and operational definitions provided in the theoretical framework of the study. Further on, the coding procedure was performed by both authors and an independent trained researcher. All instances of disagreement were resolved through consensus.

### Data Analysis

Analyses were conducted by using Comprehensive Meta-Analysis software, version 2.2.050 (Biostat Inc., Englewood, NJ, USA). As an indicator of effect sizes, Pearson’s coefficient of correlation (*r*) was used, with values above 0.50 considered large, around 0.30 considered moderate and values around 0.10 interpreted as small effects (Cohen, 1988).

Given the heterogeneity of the studies, all analyses were based on a random effects model.

### Publication Bias Analysis

To assess the risk of publication bias for the results of the meta-analysis we calculated the Begg and Mazumdar’s rank correlation test in line with the recommendations of Kepes et al. (2012). This test computes the rank order correlation (Kendall’s tau b) between the treatment effect and the standard error (which is driven primarily by sample size) in order to identify if large studies tend to be included in the analysis regardless of their treatment effect, whereas small studies are more likely to be included when they show a relatively large treatment effect.

The Begg and Mazumdar’s rank correlation test revealed a Kendall’s tau b of 0.14, with a *p*-value of 0.410 (based on continuity-corrected normal approximation), which suggests no publication bias for the overall relation between affect and EP. Similar results were obtained in the case of PA – EP and NA – EP relation respectively, with a Kendall’s tau b of 0.83, *p*-value of 0.089 in the case of the former and a Kendall’s tau b of -0.18, *p*-value of 0.198 (based on continuity-corrected normal approximation) in the case of the latter, which suggests no publication bias.

## Results

### Overall Relation among Affective Experiences and Entrepreneurial Performance

**FIGURE 1:**
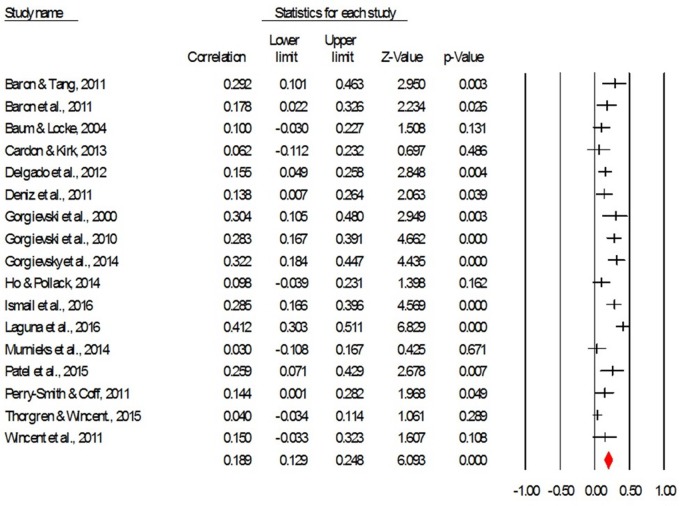
**The forest plot for the correlation between affect (both positive and negative) and EP (ignoring the direction of correlations)**.

RQ1 concerned exploring the magnitude of the relation between affect and EP. The average corrected effect size describing the correlation (ignoring the direction of correlations) between affective experiences and EP is *r* = 0.19, CI95 = [0.12, 0.24], *p* < 0.001, which represents a low to moderate effect size (Cohen, 1988). **Figure 1** provides a forest plot with the effect sizes of the studies included in the meta-analysis.

### Analysis of the Positive Affect – Entrepreneurial Performance Relation

**FIGURE 2:**
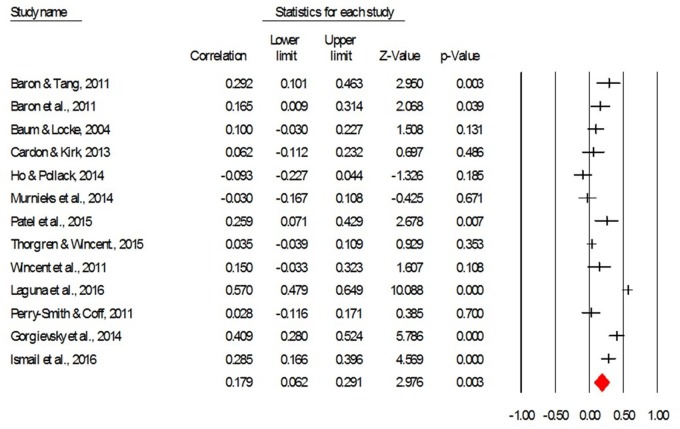
**The forest plot for the correlation between positive affect and entrepreneurial performance (taking into account the direction of correlations)**.

Positive affect was measured in 13 studies, with a total of 2808 participants. As this section concerns the relation between affect with the same valence (i.e., positive) and EP, the analyses we performed included the sign on the correlations, as reported in the original studies. The average corrected effect size describing the correlation between PA and the EP is *r* = 0.18, CI95 = [0.06, 0.29], *p* < 0.01, which represents a low to moderate effect size (Cohen, 1988). Therefore, Hypothesis 1 received support. **Figure 2** provides a forest plot with the effect sizes of the studies included in this part of the analysis.

The distribution of effects proved to be significantly heterogeneous, *Q*(12) = 114.79, *p* < 0.001, therefore we continued with performing the moderators’ analysis.

### Moderators of Effects for the Positive Affect – Entrepreneurial Performance Relation

#### Performance Dimension as a Moderator

Studies (*k* = 5) which measured the outcome variable at the individual level (i.e., innovative behavior, goal attainment etc.) obtained a significant moderate effect size, *r* = 0.30, CI_95_ = [0.13, 0.46], while studies (*k* = 11) which measured entrepreneurial outcomes related to business (i.e., sales, profit etc.) generated a significant low effect size *r* = 0.15, CI_95_ = [0.03, 0.27]. Although the effect size computed for the PA – EP at the individual level is double compared to the effect size for the PA – EP at the business level, the differences between the two categories are not significant, QB(1) = 2.15, *p* = 0.142. Therefore, Hypothesis 3a did not receive support.

#### Moderators Related to Affective Experiences

##### Status/duration of affect

Hypothesis 4a stated that the positive association between PA and EP will be stronger for trait PA as compared to state PA and it failed to receive empirical support. When studies measured state PA, the average effect size obtained was a significant small to moderate one, *r* = 0.18, CI95 = [0.03, 0.31], similar to the effect size obtained in the case of trait PA, *r* = 0.17, CI95 = [-0.05, 0.38], with no significant differences between the two conditions, QB(1) = 0.01, *p* = 0.971 (**Table 2**).

**Table 2:** Moderators of the positive affect – entrepreneurial performance effect size related to affect characteristics.

Moderator	Categories of the moderator	No of studies	Correlation *r*	Lower limit	Upper limit	QB	df	*p*
Status/duration	State	9	0.18	0.03	0.31	0.01	1	0.971
	Trait	4	0.17	-0.05	0.38			
Integrality	Incidental	5	0.22	0.03	0.40	0.38	1	0.537
	Integral	8	0.15	-0.01	0.29			


*df, degrees of freedom; *p*, significance level.*

##### Integrality of affect

Both types of PA yielded significant effect sizes, with a significant small to moderate effect for incidental affect, *r* = 0.22, CI95 = [0.03, 0.40] and a non-significant small effect for integral affect, *r* = 0.15, CI95 = [-0.01, 0.29]. The differences between the two conditions were not significant, QB(1) = 0.38, *p* = 0.537 and Hypothesis 5a did not receive support.

#### Moderators Related to the Study Quality

##### Type of measurement of the entrepreneurial performance

The analysis of the relationship between PA and EP as a function of the way EP was measured revealed that when EP was measured objectively, the effect size was significant but rather small, *r* = 0.12, CI95 = [0.01, 0.24]. On the other hand, studies with subjective measurements of entrepreneurial variables recorded a significant moderate effect size, *r* = 0.27, CI95 = [0.13, 0.41]. The comparison between the two conditions proved that even if subjective measures recorded a two times higher effect size than objective measures, the difference was only marginally significant, QB(1) = 2.65, *p* = 0.104 (RQ2) (**Table 3**).

**Table 3:** Moderators of the positive affect – entrepreneurial performance effect size related to study design characteristics.

Moderator	Categories of the moderator	No of studies	Correlation *r*	Lower limit	Upper limit	QB	df	*p*
Measurement of entrepreneurial performance	Objective	10	0.12	0.01	0.24	2.65	1	0.104
	Subjective	6	0.27	0.13	0.41			
Ecological validity	Ecological	12	0.18	0.06	0.30	0.46	1	0.495
	Non-ecological	1	0.03	-0.38	0.43			


*df, degrees of freedom; *p*, significance level.*

##### Ecological validity of the design

The analysis shows that ecological studies yielded a significant small to moderate effect size, *r* = 0.18, CI_95_ = [0.06, 0.30], while non-ecological studies obtained a non-significant effect size, *r* = 0.03, CI_95_ = [-0.38, 0.43], with no significant differences between the two categories, QB(1) = 0.46, *p* = 0.495 (RQ2).

#### Moderators Related to Individual Differences

##### Gender differences

Among the studies which measured PA, 11 reported the proportion of women. We performed a meta-regression analysis in order to test the predictive value of the proportion of women in the samples upon the effect sizes. The results revealed that this variable had a significant positive predictive value, *B* = 0.004, *p* < 0.001 (**Table 4**). In other words, larger proportions of women in the samples are associated with higher correlations between PA and EP. Hypothesis 6a received empirical support.

**Table 4:** Moderators of the positive affect – entrepreneurial performance effect size related to individual differences.

Moderator	Categories of the moderator	No of studies	Correlation *r*	Lower limit	Upper limit	QB	df	*p*
% of women	-	11	*B* = 0.004, CI95 = [0.002, 0.006], *p* < 0.001					
Average age in the sample	-	9	*B* = -0.066, CI95 = [-0.083, -0.049], *p* < 0.001					
% of higher education	-	4	*B* = -0.003, CI95 = [-0.006, -0.001] *p* < 0.01					


*df, degrees of freedom; *p*, significance level.*

##### Education

Among the selected studies, only 4 reported the proportion of higher educated participants. The analysis of the predictive value of the proportion of participants with higher education in each sample revealed that this variable had a significant negative predictive value, *B* = -0.003, *p* < 0.01 (Hypothesis 7a), which means that as the proportion of higher educated participants increases, the correlation between PA and EP decreases.

##### Average age in the samples

The meta-analytical regression performed on the nine studies which measured PA and reported the average age of the sample proved that it is a significant negative predictor of the effect sizes, *B* = -0.066, *p* < 0.001 (Hypothesis 8a). In other words, as participants are increasingly younger, the correlation between PA and EP becomes higher.

### Analysis of Negative Affect – Entrepreneurial Performance Relation

**FIGURE 3:**
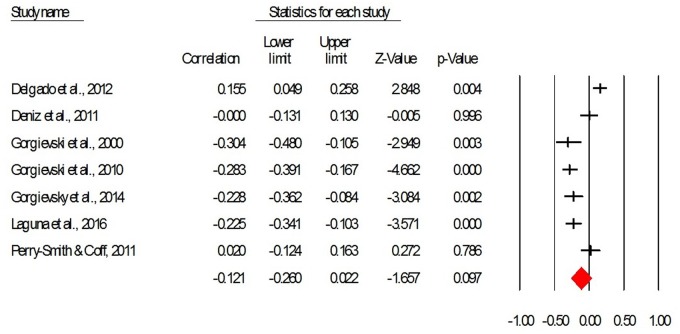
**The forest plot for the correlation between negative affect and entrepreneurial performance (taking into account the direction of correlations)**.

The relation between NA and EP was measured in seven studies, with a total of 1615 participants. As this section concerns the relation between affect with the same valence (i.e., negative) and EP, the analyses we performed included the sign on the correlations, as reported in the original studies. The average corrected effect size describing the correlation between NA and the EP is not statistically significant, *r* = -0.12, CI95 = [-0.26, 0.02], *p* = 0.097. Therefore, Hypothesis 2 stating a negative relation between NA and EP, did not receive empirical support. **Figure 3** provides a forest plot with the effect sizes of the studies included in the meta-analysis.

Even if the overall effect of NA is actually null, the distribution of effects proved to be significantly heterogeneous, Q(6) = 47.15, *p* < 0.001, which means that the moderators analysis could reveal certain categories of studies with non-null effects.

### Moderators for the Negative Affect – Entrepreneurial Performance Relation

#### Performance Dimension as a Moderator

Both studies (*k* = 4) that measured EP at the individual level (i.e., innovative behavior, goal realization etc.) and those (*k* = 4) which measured EP as a business level indicator (i.e., sales, profit etc.) obtained a non-significant effect size: *r* = -0.04, CI_95_ = [-0.26, 0.18] for the former and *r* = -0.07, CI_95_ = [-0.15, 0.29] for the latter, with no significant differences between the two categories, QB(1) = 0.50, *p* = 0.477 (Hypothesis 3b).

#### Moderators Related to Affect

##### Status/duration of affect

When studies measured state NA, the average effect size obtained was a significant negative one, *r* = -0.16, CI95 = [-0.27, -0.05], while a non-significant effect size was obtained in the case of trait NA, *r* = 0.15, CI95 = [-0.11, 0.40], with significant difference between the two conditions, QB(1) = 4.76, *p* = 0.029 (Hypothesis 4b received support) (**Table 5**).

**Table 5:** Moderators of the negative affect – entrepreneurial performance effect size related to affect characteristics.

Moderator	Categories of the moderator	No of studies	Correlation *r*	Lower limit	Upper limit	QB	df	*p*
Status/duration	State	6	-0.16	-0.27	-0.05	4.76	1	0.029
	Trait	1	0.15	-0.11	0.40			
Integrality	Incidental	5	-0.12	-0.30	0.05	0.01	1	0.898
	Integral	2	-0.10	-0.38	0.18			


*QB, the homogeneity test; df, degrees of freedom; *p*, significance level.*

##### Integrality of affect

Both types of affect yielded non-significant effects sizes, with an *r* = -0.12, CI95 = [-0.30, 0.05] for incidental emotions, and an *r* = -0.10, CI95 = [-0.38, 0.18] for integral emotions, with no significant differences between the two conditions, QB(1) = 0.01, *p* = 0.898 (Hypothesis 5b did not receive support).

#### Moderators Related to the Study Quality

##### Type of measurement for the entrepreneurial performance

The analysis of the relationship between NA and EP as a function of the way the outcome was measured revealed a non-significant result with an effect size of *r* = -0.19, CI95 = [-0.41, 0.05] for objective measurement and a significant one of *r* = -0.20, CI95 = [-0.33, -0.06] for subjective measurement of EP, with no significant differences between the two conditions, QB(1) = 0.01, *p* = 0.938 (RQ2) (**Table 6**).

**Table 6:** Moderators of the negative affect – entrepreneurial performance effect size related to study design characteristics.

Moderator	Categories of the moderator	No of studies	Correlation *r*	Lower limit	Upper limit	QB	df	*p*
Measurement of entrepreneurial variable	Objective	2	-0.19	-0.41	0.05	0.01	1	0.938
	Subjective	6	-0.20	-0.33	-0.06			
Ecological validity	Ecological	6	-0.14	-0.30	0.01	0.56	1	0.454
	Non-ecological	1	0.02	-0.36	0.40			


*df, degrees of freedom; *p*, significance level.*

##### Ecological validity of the design

The analysis of effect sizes based on the ecological nature of the design revealed non-significant results, with an effect size of *r* = -0.14, CI_95_ = [-0.30, 0.01] for ecological studies and *r* = 0.02, CI_95_ = [-0.36, 0.39] for non-ecological studies, with no significant differences between the two categories, QB(1) = 0.56, *p* = 0.454 (RQ2).

#### Moderators Related to Individual Differences

##### Gender

We performed a meta-regression analysis in order to test the predictive value of gender upon the effect sizes on the data from the four studies that reported the proportion of women. The results revealed that this variable had a significant positive predictive value, *B* = 0.002, *p* < 0.001 (**Table 7**). In other words, larger proportions of women in the samples are associated with higher correlations between NA and EP. As a consequence, Hypothesis 6b received empirical support.

**Table 7:** Moderators of the negative affect – entrepreneurial performance effect size related to individual differences.

Moderator	Categories of the moderator	No of studies	Correlation *r*	Lower limit	Upper limit	QB	df	p
% of women	–	6	*B* = 0.002, CI95 = [0.001, 0.003], *p* < 0.001					
Average age of the sample			*B* = -0.025, CI95 = [-0.036, -0.015], *p* < 0.001.					


*df, degrees of freedom; *p*, significance level; CI, confidence interval.*

##### Education

Education could not be analyzed as a predictor of the effect size (Hypothesis 7b) because there were only two studies that measured NA and also reported the proportion of higher educated participants in the sample, and meta-regression cannot be performed on less than three studies.

##### Average age in the samples

The meta-analytical regression performed on the five studies which measured NA and reported the average age of the sample proved that it is a significant negative predictor of the effect sizes, *B* = -0.025, *p* < 0.001 (Hypothesis 8b). In other words, as participants are increasingly younger, the correlation between NA and EP becomes higher.

## Discussion

Our meta-analysis had three important aims: (1) to explore the magnitude of the affect – EP relation, (2) to explore the differential impact of PA and NA respectively on EP, and (3) to test the moderating role of a set of theoretical and methodological factors on the PA – EP and the NA – EP relations.

With respect to our first objective, entrepreneurship research has so far postulated the existence of a rather strong connection between affect in general and the entrepreneurial process, mostly due to the high personal stakes that the entrepreneur throws to the game and to the complex endeavors s/he faces during the entrepreneurial process (entrepreneurial tasks are new, intricate, and described by uncertainty) (Baron, 2008; Doern and Goss, 2013; Foo et al., 2015; Delgado Garcia et al., 2015). In this sense, entrepreneurship is traditionally considered ‘hot’ or an *emotional journey* (Baron, 2008; Cardon et al., 2012). However, the quantitative analysis of the effect sizes included in the meta-analysis recommends a shift of perspective. Overall, the results show a low to moderate effect size for the affect (including PA and NA variables) – EP relation (when ignoring the correlation signs) (*r* = 0.17, *p* < 0.001). In this light, entrepreneurship is rather ‘cold.’ The reported magnitude of the affect – EP is similar or below to effect sizes reported in other meta-analyses on emotional factors and various outcomes (i.e., decision making, in-role or extra-role performance, creativity etc.) (Lyubomirsky et al., 2005; Kaplan et al., 2009; Angie et al., 2011).

Our second goal concerned exploring the differential association between PA and EP, on the one hand, and NA and EP, on the other hand (taking into consideration the sign of the correlations). While the evidence from previous entrepreneurship research supporting a positive association between PA and EP was quite robust, there were also instances when PA was reported to have a detrimental effect on various measures of EP (e.g., Baron et al., 2011). The results of our meta-analysis shed light over such contradictory findings and indicate a positive and significant association between PA and EP outcomes such as: innovation, sales, venture growth, goal attainment etc. (*r* = 0.17, *p* < 0.001). This is in line with Fredrickson’s (2001) broaden-and-build theory that states that PA signals a benign environment that further encourages the entrepreneur to broaden her/his attention scope and invest more in exploring, creating and seizing opportunities. A similar explanation is derived from the approach-avoidance theories (Gray and McNaughton, 2000; Corr, 2013). In short, PA is considered to activate the BAS, an underlying neuropsychological system that triggers appetitive, reward seeking behaviors, which are aligned to the specifics of entrepreneurial tasks and conducive for performance. Future research could explore additional affective dimensions such as different types of appraisal or the influence of emotion regulation strategies and other contextual contingencies in order to better understand the nature of the PA – EP relation and the previous inconsistencies.

On the other hand, contrary to our expectations, in this meta-analysis we found that, overall, NA had no significant (negative) implications for EP (*r* = -0.12, *p* = 0.097). This is surprising, since scholars have traditionally argued toward a more significant effect of NA on entrepreneurial processes. One argument concerned the increased frequency and intensity of negative affective experiences encountered during the entrepreneurial process (Markman et al., 2002; Doern and Goss, 2014). The other one claimed that NA has a higher influence on several psychological processes, as compared to PA (Baumeister et al., 2001). However, this non-significant should be approached with prudence due to the low number of studies that included measures for the NA-EP relation.

All in all, while negative moods, emotion and affective dispositions don’t seem to matter for the extent to which a company attains goals such as profitability, business growth and innovation, positive emotions, moods and dispositions prove to be beneficial. The *happy-worker-productive worker* metaphor can thus become the *happy entrepreneur – successful venture* metaphor.

For a comparison, the overall effect of PA on EP is similar to the effect of personality traits on business creation (*r* = 0.19) and entrepreneurial success (*r* = 0.195) (Rauch and Frese, 2007), or that of entrepreneurial social capital on business performance (*r* = 0.211) (Stam et al., 2014), stronger than the effect of human capital over entrepreneurial success (*r* = 0.098) (Unger et al., 2011), and lower than the effect of entrepreneurial orientation on business performance (*r* = 0.242) (Rauch et al., 2009).

### Effects of Moderating Variables on the PA – EP and NA – EP Associations

The following section reviews the results from the moderator analysis (i.e., the third aim of the paper), investigating how variables pertaining to the level of analysis for the outcome, to affect characteristics, individual differences and study quality influence the relation between PA and EP and NA and EP, respectively.

Apparently, the magnitude of the reported effect sizes for the PA – EP relations do not change significantly as a function of the level of measurement for the outcome (i.e., individual vs. company level EP), nor as a function of affect characteristics, such as duration (state vs. trait affect) or integrality (whether affect is incidental or integral to the entrepreneurial task). The results are similar for the NA – EP relation, with one exception concerning the duration of affect. In particular, state NA is significantly and negatively related to EP, as compared to trait NA, which has no significant effect. This means that entrepreneurs experiencing negative state affect are likely to report lower EP outcomes, while trait negative affect does not seem to have any significant implication (i.e., contrary to our expectations). These results should be interpreted with caution due to the limited number of studies exploring the NA-EP relation (i.e., one study for trait NA and six studies for state SA). However, an alternative explanation for these results could be derived from the Affect Events Theory (i.e., AET, Weiss and Cropanzano, 1996). AET argues that state affect plays a mediator role in the relation between trait affect and various outcomes and, thus, it is likely to be more intimately linked with EP.

As far as the individual differences are concerned, the results show gender and age are influential for both PA-EP and NA-EP relations, while education is influential only for the PA-EP association. For instance, the results of our meta-analysis show that women entrepreneurs are more vulnerable to the influence of PA and NA. Women entrepreneurs who experience PA are likely to report more positive outcomes related to EP, while those experiencing NA are likely to report less satisfying EP indicators. This vulnerability is in line with the findings from the general emotion and neuroanatomy literature which illustrate that women show greater emotional reactivity than men (Bradley et al., 2001; Wild et al., 2001). As far as education and age are concerned, as we expected, as the proportions of higher educated or more aged participants increase, PA tends to have a slightly lower influence over EP. In a similar vein, as the proportion of more aged entrepreneurs increases, NA tends to have a less strong negative connection to EP. One plausible explanation could be related to the maturation of pre-frontal cortical regions which are implicated in active emotion regulation strategies (Martin and Ochsner, 2016) that downplay the influence of affect on various cognitive and motivational processes. On the other hand, for the NA – EP relation, the moderating role of education could not be tested due to the low number of studies that reported such demographics.

In this meta-analysis, we also tested the moderating role of study quality on the PA – EP and NA – EP relations, via two underlying moderators: the type of measurement for the outcome variable (objective vs. subjective) and to what extent the original studies employed an ecological design. Our results show that there are no significant variations in the effect sizes pertaining to the PA-EP and NA-EP relations according to the ecological quality of the design. Researchers can thus employ vignettes and simulations (coded as non-ecological in our meta-analysis) in studying affect – performance research questions, without any detrimental effects. The type of measurement for the outcome variable did not reach classical significance levels (*p* = 0.104) for the PA-EP relation. However, by exploring the trends, when EP was measured objectively, the effect size for PA was significant but rather small, whereas when it was measured subjectively the effect size was significant and moderate. This finding recommends a careful approach to choosing EP measures in future research, so as to eliminate the overestimation effect due to the measurement method employed.

### Limitations and Future Research

Next to the contributions, this study also has some limitations. Our analyses revealed a positive association between PA and EP, and a negative relation only between state NA and EP. As previously argued, the lack of significance for the trait NA-EP association should be interpreted with caution, due to the reduced number of studies exploring this relation (only one in this meta-analysis). Future research in the field of entrepreneurship could allocate more interest to the role of negative affect for EP in order to shed light on the true relation between them. Considerable less studies explored the relation between negative affective states and EP, revealing a “positivity bias” in entrepreneurship research. Further on, future research could explore the role of mixed emotions on EP outcomes, as the emotional life of entrepreneurs is rarely homogenous. In addition, more focus is needed on exploring the mechanisms through which entrepreneurial affect drives company level performance outcomes. The influence of entrepreneurial affect on employees’ affect and motivation, via emotional contagion, mimicry and other mechanisms, and the subsequent link with EP remains an interesting avenue for future exploration.

The moderator analysis regarding affect characteristics yielded no difference in the magnitude of the effect for incidental or integral affect, nor for state vs. trait affect. In line with Delgado Garcia et al. (2015), we argue that future research might explore other dimensions of affective experiences such as different patterns of appraisal (i.e., control, certainty etc.) or meta-emotional abilities (i.e., emotion regulation strategies) in order to more fully understand the emotional journey of entrepreneurs.

With respect to methodology, although marginal in terms of statistical significance, the results point out a tendency toward a slightly inflated effect of PA on EP, when subjective measures are employed for the outcome variable. Therefore, in order to shed light upon the true magnitude of the relation, future studies should employ more objective measures of EP related variables. In addition, we call for a more rigorous reporting of the individual differences that make up the samples included in the studies, such as participants’ gender, age, education level, experience, type of entrepreneur (serial vs. one-shot), culture and industry. On the one hand, these variables were empirically and/ or just theoretically proved to be important moderators of the affect – EP relation. On the other hand, as previously mentioned, the influence of some of these sample characteristics could not be empirically estimated due to insufficient data reported in the original studies (as in the case of type of entrepreneurs, culture, industry etc.).

Also, as in any other similar endeavors, the quality of the meta-analysis is not independent from the quality of the primary studies included in the analysis. For instance, the causal link between affective experiences and the EP has been rarely addressed in its stricter sense, as most of this area of research relies on cross-sectional or qualitative studies. In fact, with two exceptions (i.e., longitudinal studies), the majority of the studies included in this meta-analysis were cross-sectional, therefore the issue of reversed causality in the PA-EP and NA-EP relation cannot be ruled out at this point. Future studies aiming to test causal inferences could benefit more from experimental designs. Intensive data collection designs such as experience sampling methodology (ESM) could also enrich our understanding of the affect – EP relations, by exploring the within individual dynamics. ESM also improves the ecological validity of results, and minimizes retrospective biases (Uy et al., 2010).

## Conclusion

By synthesizing the current findings in the entrepreneurship literature on the association between PA and EP and NA and EP, this meta-analysis provides a useful insight on the implications of entrepreneurial affect for attaining goals such as profitability, business growth and innovation. This is a departure from other meta-analyses exploring affect and individual (not business) performance in more general settings. In particular, our results show that entrepreneurial PA is positively related to EP, while only state and not trait NA has a significant negative association with EP. These results vary especially in relation with individual differences such as gender, age, and education, with women, younger and less educated (education is a moderator only in the case of PA-EP relation) entrepreneurs displaying stronger associations between PA and EP and NA and EP. Practical implications can be derived from the field of positive psychology, concerning interventions that aim to increase the experience of PA within the entrepreneurial context and down-regulate NA in order to foster EP.

## Author Contributions

OF and SP were involved in study design, data collection and analysis, writing and editing the manuscript.

## Conflict of Interest Statement

The authors declare that the research was conducted in the absence of any commercial or financial relationships that could be construed as a potential conflict of interest.
